# Anti-tau conformational scFv MC1 antibody efficiently reduces pathological tau species in adult JNPL3 mice

**DOI:** 10.1186/s40478-018-0585-2

**Published:** 2018-08-22

**Authors:** Francesca Vitale, Luca Giliberto, Santiago Ruiz, Kristen Steslow, Philippe Marambaud, Cristina d’Abramo

**Affiliations:** Litwin-Zucker Center for Research in Alzheimer’s Disease, The Feinstein Institute for Medical Research, Northwell Health, 350 Community Drive, Manhasset, NY 11030 USA

## Abstract

Tau, the main component of the neurofibrillary tangles (NFTs), is an attractive target for immunotherapy in Alzheimer’s disease (AD) and other tauopathies. MC1/Alz50 are currently the only antibodies targeting a disease-specific conformational modification of tau. Passive immunization experiments using intra-peritoneal injections have previously shown that MC1 is effective at reducing tau pathology in the forebrain of tau transgenic JNPL3 mice. In order to reach a long-term and sustained brain delivery, and avoid multiple injection protocols, we tested the efficacy of the single-chain variable fragment of MC1 (scFv-MC1) to reduce tau pathology in the same animal model, with focus on brain regional differences. ScFv-MC1 was cloned into an AAV delivery system and was directly injected into the hippocampus of adult JNPL3 mice. Specific promoters were employed to selectively target neurons or astrocytes for scFv-MC1 expression. ScFv-MC1 was able to decrease soluble, oligomeric and insoluble tau species, in our model. The effect was evident in the cortex, hippocampus and hindbrain. The astrocytic machinery appeared more efficient than the neuronal, with significant reduction of pathology in areas distant from the site of injection. To our knowledge, this is the first evidence that an anti-tau conformational scFv antibody, delivered directly into the mouse adult brain, is able to reduce pathological tau, providing further insight into the nature of immunotherapy strategies.

**Electronic supplementary material:**

The online version of this article (10.1186/s40478-018-0585-2) contains supplementary material, which is available to authorized users.

## Introduction

In Alzheimer’s disease (AD), neurofibrillary pathology positively correlates with cognitive decline, emphasizing the direct link between pathological tau accumulation and neurodegeneration [8, 9, 30, 39, 50]. Several studies show efficient reduction of tau pathology in transgenic animal models, using an immunotherapeutic approach, with different yields depending on the targeted epitopes [3, 6, 7, 12, 14, 16, 17, 45, 52, 53]. Indeed targeting total tau, tau phosphorylation or conformational epitopes might result in different outcomes, in terms of efficiency and safety.

Tau conformational change, targeted by the MC1 antibody, is one of the earliest detectable events in the brain of AD patients. MC1 and Alz50 are the only tau antibodies targeting the AD-specific epitope formed by two discontinuous portions of tau, _7_EFE_9_ and _313_VDLSKVTSKC_322_ [21, 28, 29]. This aberrant conformation of tau was shown to be present in a soluble form of the protein and in paired helical filaments (PHF) assemblies [47]. Importantly, the level of MC1 reactivity correlates with the severity and progression of AD [27]. From a therapeutic perspective targeting this tau structural modification is a very attractive approach to pursue. In conventional passive immunotherapy studies performed in mice, we and others [14, 17] have previously shown that targeting the MC1 epitope can efficiently reduce neurofibrillary pathology in forebrain, highlighting the importance of tau epitope specificity: the ability to discriminate between normal tau and pathological tau species confers MC1 a remarkable advantage as immunotherapeutic tool compared to pan-tau and phospho-tau antibodies, which on the contrary might interfere with the normal function of tau. Of note, humanized MC1 (LY3303560) has recently entered a Phase II study to treat early symptomatic Alzheimer’s disease [ClinicalTrials.gov, accession number NCT03518073].

The main challenge in neuro-therapeutic development in humans is a successful delivery of molecules into the brain parenchyma. The first obstacle in this process is crossing the blood brain barrier (BBB) and achieving widespread brain diffusion of the drug [40]. In addition to low tissue/cell penetration, using whole monoclonal antibodies (mAbs) might result in potential serious adverse effects such as inflammatory reactions and cerebral microhemorrhages [5, 44, 49]. Finally, the relative short half-life of conventional mAbs poses a question of long term sustainability of such treatments, with need for repeated infusions, and issues of compliance and cost. Hence, the need to develop new and safer tools for passive tau immunotherapy.

Antibody engineering represents an important alternative approach to increase brain penetration, while limiting the deleterious effects of an uncontrolled immune response. Most recently, a study [32] showed that, in vivo and in cultured neurons, antibody effector function (i.e. Fc region) is not required for targeting and clearing tau with specific mAbs; reducing the effector function may offer a safer approach for targeting tau by avoiding engagement of microglia that may induce an inflammatory response but still achieving clearance of pathological tau. Furthermore, a recent study using AAV-vectored intracerebral passive immunization with the anti-phospho-tau monoclonal antibody PHF1 (tau pSer396/404) [35] was proven efficacious in adult P301S mice in reducing insoluble pathological phosphorylated tau (p-tau) in the hippocampus, with some reduction of p-tau immunoreactivity in the cortex. Also, an anti-pan-tau single chain variable fragments antibody (scFv) [26] was shown to reduce soluble tau pathological species in specific hippocampal regions in 9 month old mutant P301S injected at birth. In line with these findings, we have engineered MC1 as scFv to target tau in the brain of adult JNPL3 mice. ScFv are the smallest antibody fragments containing a complete antigen-binding site, consisting of the light and heavy-chain variable domains covalently joined by a polypeptide linker and lacking the Fc region [2, 4, 25, 51]. In order to sustain the expression of antibody fragments over long periods, scFv-MC1 has been cloned in the adeno-associated viral vector serotype 5 (AAV5) and delivered by a one-time intracranial injection [10, 11, 19, 23, 24, 33]. Here we show that scFv-MC1 is actively expressed and released in the extracellular milieu upon AAV5-vectored hippocampal injection, exerting its effect also in areas distant from the site of injection. Moreover, in this system we are able to target either neurons or astrocytes, and we show that the astrocytic machinery works more efficiently at reducing soluble, oligomeric and insoluble tau species in different brain areas.

## Materials and methods

### ScFv-MC1 design, expression and purification

**Fig. 1:**
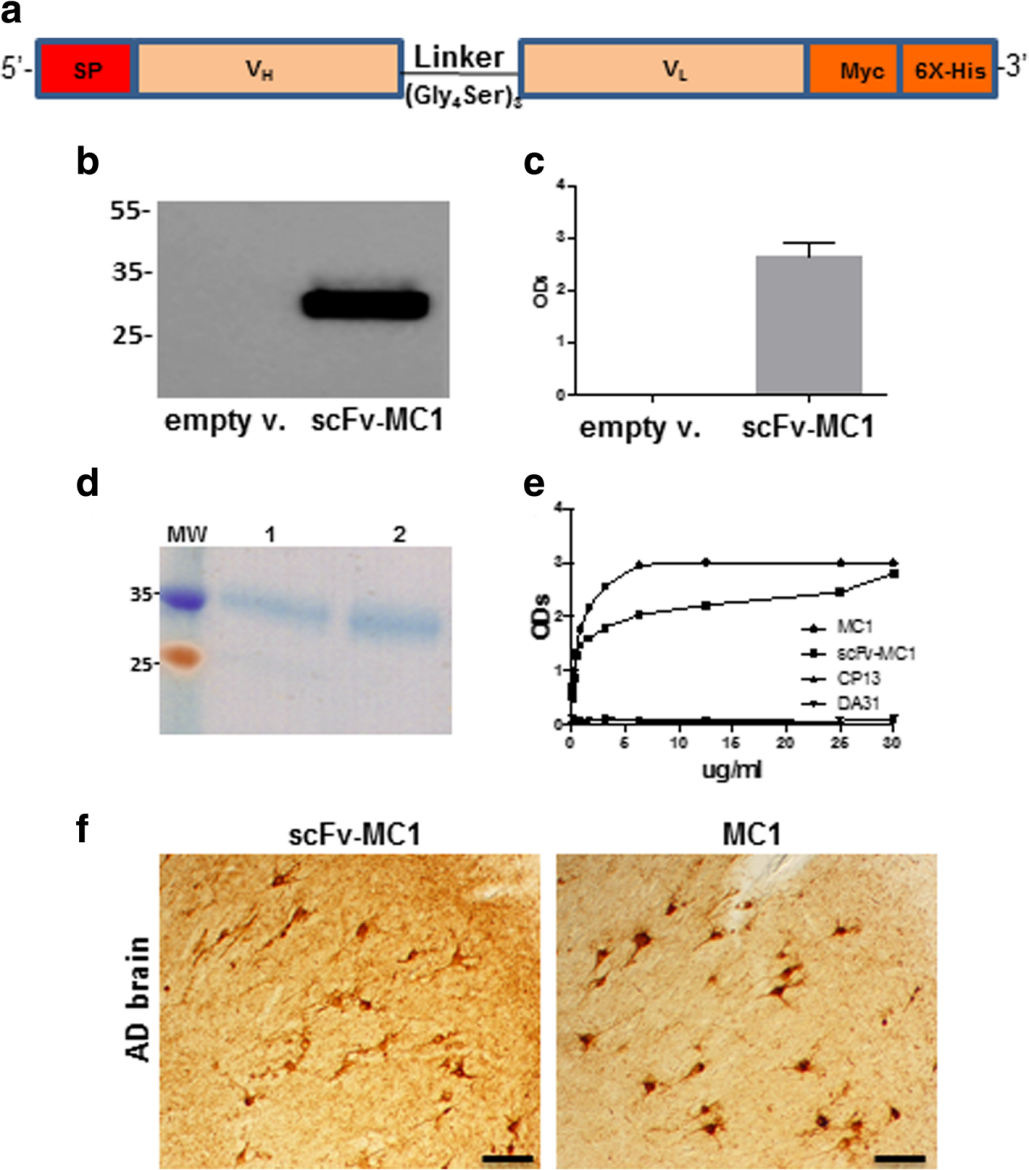
ScFv-MC1 design, expression and characterization. **a** scFv-MC1 diagram: 5’-terminal signal peptide (SP), variable heavy (V_H_) and variable light (V_L_) chains joined together by a 15 amino acid residue linker (Gly_4_Ser)_3_, and 3’-terminal Myc and His6X tags. **b** HEK293 cells were transiently transfected with empty vector (empty v.) or scFv-MC1. In the cell lysate (immunoblotting) the scFv-MC1 was detected as a 30 kDa band; **c** in the conditioned medium the presence of scFv-MC1 was assessed using a specific ELISA and was expressed as ODs (optical densities). Cells transfected with the empty vector did not show any sign of reactivity in western blotting or ELISA. **d** Purified scFv-MC1, run on SDS-PAGE gel and stained with Coomassie: two different purified preparations (1, 2) revealed the expected molecular weight (30 kDa). **e** scFv-MC1 antigen-binding specificity: purified scFv-MC1 tested for its activity on the specific peptide (998b) was compared to the MC1 mAb, showing a comparable reactivity; CP13 and DA31 were used as negative controls. **f** Representative images of AD brain immunohystochemistry: both scFv-MC1 and MC1 (1:500 dilution) showed specificity for the neurofibrillary pathology (Olympus BH-2 microscope, bar: 50 μm)

The MCLAB antibody service (San Francisco, CA) was employed in order to sequence the light and heavy-chain variable domains corresponding to the MC1 antibody. The service provides RNA extraction from hybridoma cell pellets and transcription into cDNA, followed by amplification of the heavy and light chains using degenerate variable heavy (V_H_) and variable light (V_L_) chains primers. The V_H_ and V_L_ chains were joined together by a 15 amino acid residues linker (Gly_4_Ser)_3_. 5’-terminal signal peptide (SP) and 3’-terminal Myc and His6X tags were added (Fig. 1a). ScFv-MC1 was cloned into the mammalian expression vector pcDNA3.1 (Genewiz, South Plainfield, NJ). HEK293T were seeded 24 h prior to transfection on 6-well plates, at a density of 400,000 cells/well. Lipofectamine 2000 (Invitrogen, Carlsbad, CA) was used for transfection, with 0.5 μg of pcDNA3.1 encoding scFv-MC1. After 48 h, the scFv-MC1 released into the conditioned medium was affinity purified using a Ni-Sepharose High Performance column (GE Healthcare, Port Washington, NY). The efficiency of purification was tested using the same immunosorbent assay employed to assess the antigen–binding specificity of the scFv (described below). Starting material, flow through and eluted fractions were tested to check for proper enrichment of the purified material. The purified scFv-MC1 was checked on Coomassie-stained SDS-PAGE gel for proper molecular weight.

### ScFv-MC1 antigen-binding specificity

The scFv antigen-binding reactivity was measured using an immune-sorbent assay where 96-well plates were coated over-night (O/N) with Neutravidin (Sigma-Aldrich, Saint Louis, MO), followed by incubation with the MC1 specific 998-biot peptide (GenScript, Piscataway, NJ). Purified scFv-MC1, MC1, DA31 and CP13 (negative controls) were diluted in 5% non-fat milk and added to the wells. After incubating for 1 h at room temperature (RT), mouse anti-His6Xtag Ab was added (1:1000 dilution) (Thermo Fisher Scientific, Waltham, MA) followed by goat anti-mouse IgG-HRP (1:1000 dilution) (SouthernBiotech, Birmingham, AL). Bio-Rad HRP Substrate Kit (Bio-Rad laboratories, Hercules, CA) was used for the detection and plates were read with an Infinite m200 plate reader (Tecan, San Jose, CA) at 415 nm.

### Adeno-associated viral vector (AAV) serotype and cellular selectivity

The AAV packaging and purification service was provided by Vector Biolab (Malvern, PA). ScFv-MC1 was sub-cloned into the adeno-associated viral vector serotype 5 (AAV5) under the control of either the synthetic strong CAG (CMV-chicken beta actin-rabbit beta globin) or the GFAP (glial fibrillary acidic protein) promoter. In order to enhance expression of the transgene, the WPRE Woodchuck hepatitis virus (WPRE) post-transcriptional regulatory element was added 5’ of the Myc and His6X tags. AAV5-scFv-MC1 (CAG or GFAP promoter) or AAV5-eGFP (enhanced green fluorescent protein under CAG or GFAP control expression) was injected at a dose of 2X10^10^ GC per hemisphere.

### Transgenic mice and stereotaxic injections

JNPL3 mice obtained from Taconic (Germantown, NY) express 0N4R human tau with the P301L mutation that causes frontotemporal dementia in humans, under the mouse prion promoter. JNPL3 mice develop NFTs as early as 4.5 months and in later stages progressive deterioration of the motor function.

Intra-hippocampal injections of AAV vectors were performed according to a stereotaxic surgery protocol previously published [13]. Under sterile conditions, three month-old JNPL3 mice were anesthetized and secured on a stereotaxic frame (David Kopf instruments, Tujunga, CA). Mice received bilateral hippocampal injection of AAV preparations using a neuro syringe with a 33 gauge needle (Hamilton, Reno, NV). Injections were performed using the following coordinates: AP-2.1 from bregma, ML +/− 2.0 from bregma, DV-1.8 below dura. After injection, the needle was left in place for 5 min to minimize backflow and then slowly removed. Mice were housed individually for 2 weeks to completely recover from surgery. Animals were treated according to the current regulations for the proper handling of research animals, following an approved IACUC protocol.

Pilot injections were performed in non-transgenic animals (C57BL/6) in order to evaluate general tolerance: no weight loss or motor changes were detected at 6 weeks post-injection (*n* = 10).

Female JNPL3 mice (*n* = 18) were used to validate the AAV5-scFv-MC1 expression system in brain (injected at 3 month of age and sacrificed 4 weeks later). In addition, the efficacy study was performed on 45 females JNPL3 (n  =  15 per group): mice were injected at 3 month of age and sacrificed four months later. Overall 73 mice were employed in this study.

### Brain extracts preparation

Four months after the intracranial injection, mice were sacrificed by isoflurane overdose, decapitated and processed as described previously [17]. The brain was removed and divided at the midline so that just one half of the brain was dissected for biochemical analysis. The cortex, hippocampus and hindbrain were homogenized separately using an appropriate volume of homogenizing buffer, a solution of Tris-buffered saline (TBS), pH 7.4, containing 10 mM NaF, 1 mM Na_3_VO_4_ and 2 mM EGTA, plus the complete Mini protease inhibitor cocktail (Roche, Indianapolis, IN). Supernatants were analyzed for protein concentration using DC Protein Assay (Bio-Rad Laboratories). Brain homogenates were stored at − 80 °C and used for separate measurement of soluble and insoluble tau. Soluble tau was measured as heat-stable preparation (hsp) from brain. Hsp were prepared by adding 5% ß-Mercaptoethanol and 200 mM NaCl to the brain homogenates. Samples were then heated at 100 °C for 10 min and cooled at 4 °C for 30 min. After centrifuging at 14,000 g in a table-top microcentrifuge at 4 °C for 15 min, supernatants were collected and 5X sample buffer (Tris-buffered saline, pH 6.8 containing 4% SDS, 2% beta-mercaptoethanol, 40% glycerol and 0.1% bromophenol blue) was added. To obtain insoluble tau preparations (INS), homogenates were thawed and spun at 14,000 g for 10 min at 4 °C. The collected supernatants were centrifuged at 200,000 g for 30 min at room temperature (RT); the pellets were then re-suspended in homogenizing buffer and centrifuged again at 200,000 g for 30 min at 25 °C. The final pellets were re-suspended in 1X sample buffer and heated at 100 °C for 10 min to efficiently dissociate the insoluble tau fraction.

### Tau ELISAs

Levels of total and phosphorylated tau were assessed using the Low-tau ELISA protocol previously published [1, 22]. 96-well plates were coated for 48 h at 4 °C with specific purified monoclonal tau antibodies (DA31, CP13, PHF1, RZ3) at a concentration of 6 μg/ml. After washing, plates were blocked for 1 h at RT using StartingBlock buffer (Thermo Fisher Scientific, Waltham, MA). Brain samples and standards were diluted in 20% SuperBlock buffer (Thermo Fisher Scientific) in 1XTBS and loaded on the plates. Once the samples were added, the total tau detection antibody DA9-HRP, diluted 1:50 in 20% SuperBlock in 1XTBS, was added to the samples and tapped to combine. Plates were then incubated overnight at 4 °C. Next day, 1-Step ULTRA TMB-ELISA (Thermo Fisher Scientific) was added for 30 min at RT, followed by 2 M H_2_SO_4_ to stop the reaction. Plates were read with Infinite m200 plate reader (Tecan, San Jose, CA) at 450 nm.

### Immunoblotting

An aliquot of the total lysates was used for immunoblotting. 0.1% SDS was added to the lysates, followed by sonication (3 cycles, 10 s each). Samples were run on 4–20% Criterion Tris-HCl gels (Bio-Rad Laboratories) and electrophoretically transferred to a nitrocellulose membrane (Thermo Fisher Scientific). Residual protein-binding sites were blocked by incubation with 5% non-fat milk in 1XTBST (1X TBS plus 0.1% Tween 20) 1 h at RT, followed by an O/N incubation at 4 °C with primary antibodies diluted in 20% SuperBlock buffer (Thermo Fisher Scientific) in 1XTBST. Mouse anti-N-Cadherin A60 (BD, Franklin Lakes, NJ), mouse anti-actin Ab5 (BD) were diluted 1:5000; anti-Myc-tag 9B11 (Cell Signaling, Danvers, MA) was diluted 1:1000. Appropriate isotypes secondary antibodies HRP-conjugated were diluted 1:2000 in 5% non-fat milk 1XTBST, and added for 1 h at RT. Every step was followed by 3 or 4 washes in 1X TBST. Detection was performed using Pierce ECL Western Blotting Substrate (Thermo Fisher Scientific) or SuperSignal West Dura extended duration substrate (Thermo Fisher Scientific) and exposed to x-ray films.

### Immunocytochemistry, immunofluorescence and image analysis

Tau staining [17] and immunofluorescence were performed according to standardized protocols. After decapitation, half of the brain was fixed overnight in 4% paraformaldehyde at 4 °C. Serial sections were cut from the fixed brain half on a vibratome, conserved in TBS (50 mM Tris, 150 mM NaCl, pH 7.6)/0.02% NaN3, and stained on 24-well plates with a panel of tau antibodies and an anti-Myc-Tag (9B11) mouse mAb (Cell Signaling). Endogenous peroxidases were quenched with 3% H_2_O_2_/0.25% Triton X-100/1XTBS for 30 min. Non-specific binding was blocked with 5% non-fat milk-1XTBS for 1 h at RT. Primary antibodies were used as follows: anti tau antibodies CP13 and PHF1 (1:5000), RZ3 and MC1 (1:500) and anti-Myc-Tag (1:1000) all diluted in 5% non-fat milk-1XTBS, and incubated O/N at 4 °C, shaking. Human AD brains were stained with MC1 and scFv-MC1 both at 1:500 dilutions. Biotin-conjugated secondary antibodies (SouthernBiotech, Birmingham, AL) directed against the specific isotypes were diluted 1:1000 in 20% SuperBlock, left for 2 h at RT, and lately Streptavidin-HRP (SouthernBiotech) was incubated for 1 h. Staining was visualized by 3,3’-Diaminobenzidine (Sigma-Aldrich).

For immunofluorescence, sections were pre-incubated 5 min at RT in 1XPBS (Gibco, Carlsbad, CA) containing 0.2% TritonX100 (Sigma-Aldrich). After blocking 1 h at RT with a solution containing 5% normal goat serum (Sigma-Aldrich) diluted in 1XPBS/0.1%Triton, sections were incubated with primary antibodies diluted in 1% normal goat serum in 1XPBS/0.1% Triton: NeuN 1:1000 (EMD Millipore, Burlington, CA) MAP2 1:1000 (EDM Millipore), anti-Myc-Alexa555 1:500 (EMD Millipore), GFAP 1:1000 (Cell Signaling). After washing 3X in 1XPBS/0.1%TritonX100, Alexa Fluor secondary antibodies − 488, − 568, − 633 (Invitrogen, Carlsbad, CA) were added at 1:1000 dilutions for 1 h at RT, in different combinations in order to obtain multiple labeling images. DAPI (Invitrogen) was used to counterstain. Brains slices were then mounted on slides and let dry 20 min before being cover-slipped using Vectashield hard set anti-fade mounting (Vector Laboratories, Burlingame, CA).

Images were acquired using Olympus BH-2 bright field microscope (Waltham, MA), Zeiss Axio Imager Apotome and Zeiss 880 confocal laser miscroscopes (Peabody, MA). The acquisition parameters were kept the same for all conditions and images were analyzed and processed using ImageJ/Fiji software (NIH) and Photoshop CS6 software (Adobe). Semi-quantification was done on the hippocampal quadrant CA1 and on the entorhinal cortex by using the measure particles tool, working with 8-bit images and adjusting the threshold.

### Tau and anti-scFv-MC1 antibodies detection in serum

A detailed protocol was previously published to detect total tau in serum [18]. Upon sacrifice mice were bled, samples collected and allowed to clot for 30 min at RT. After cooling for 15 min, samples were spun at 14,000 g for 10 min at 4 °C; supernatants were collected and then re-spun at 14,000 g for 5 min at 4 °C. The final supernatants correspond to the serum samples. In order to detect tau in serum, samples were diluted 1:3 in 0.2 M NaOAc, pH 5.0 and heated at 90 °C for 15 min. After the heat treatment, samples were allowed to cool at 4 °C for 15 min, and then spun at 15,000 g for 10 min. Supernatant were collected and 1 M Tris buffer was added to neutralize the pH. After diluting 1:2 in 20% Superblock, samples were loaded on the total tau ELISA (DA31 capture).

In order to detect antibodies directed to the scFv-MC1, 96-well plates were coated with purified scFv-MC1 at 6 μg/ml for at least 24 h. Plates were washed 3X and blocked for 1 h using StartingBlock (Thermo Fisher Scientific). Plates were washed 5X and 50 μl of sera added in triplicate at 1:1000 dilution in 20% SuperBlock (Thermo Fisher Scientific). After 1 h incubation plates were washed 5X and 50 μl of goat anti-mouse non-specific IgG HRP-conjugated (SoutherBiotech) was added and incubated for 1 h. Finally, Bio-Rad HRP Substrate Kit has been used for the detection and plates were read with Infinite m200 plate reader (Tecan) at 415 nm.

### Statistical analysis

Quantitative data were analyzed using the dedicated software GraphPad Prism vs6 (GraphPad software Inc., CA). One-way ANOVA was performed when the parametric assumption of normality (D’Agostino-Pearson omnibus test) was accomplished. When not, non-parametric Kruskal-Wallis was performed instead. Statistical significance was set at *P* < 0.05. Error bars represent the SEM. Linear regression analysis was performed for all correlations.

## Results

### ScFv-MC1 is efficiently secreted in vitro, and its antigen-binding specificity is comparable to the MC1 parent antibody

ScFv-MC1 was expressed in the HEK293T cell line in order to assess for efficient secretion in medium. The scFv was detected in cell lysates (Fig. 1b) and conditioned medium of the HEK293T after 48 h of transient transfection (Fig. 1c). Taking advantage of the His6X-tag added at the 3’-end of the DNA construct, the recombinant antibody was purified using a Ni-Sepharose column. Coomassie-stained SDS-PAGE gel was used to confirm molecular weight (around 30 kDa) and purity of the preparations (Fig. 1d). The scFv-MC1 antigen-binding reactivity was measured against an MC1 specific peptide (Fig. 1e) and shown to be as specific as the parent antibody. To complete its characterization, purified scFv-MC1 was employed to stain AD human brains, confirming its specificity in binding NFTs similarly to the parent MC1 antibody (1:500 dilution) (Fig. 1f).

### AAV5-scFv-MC1 transduces neurons and astrocytes in vivo, under the CAG or the GFAP promoters respectively

**Fig. 2:**
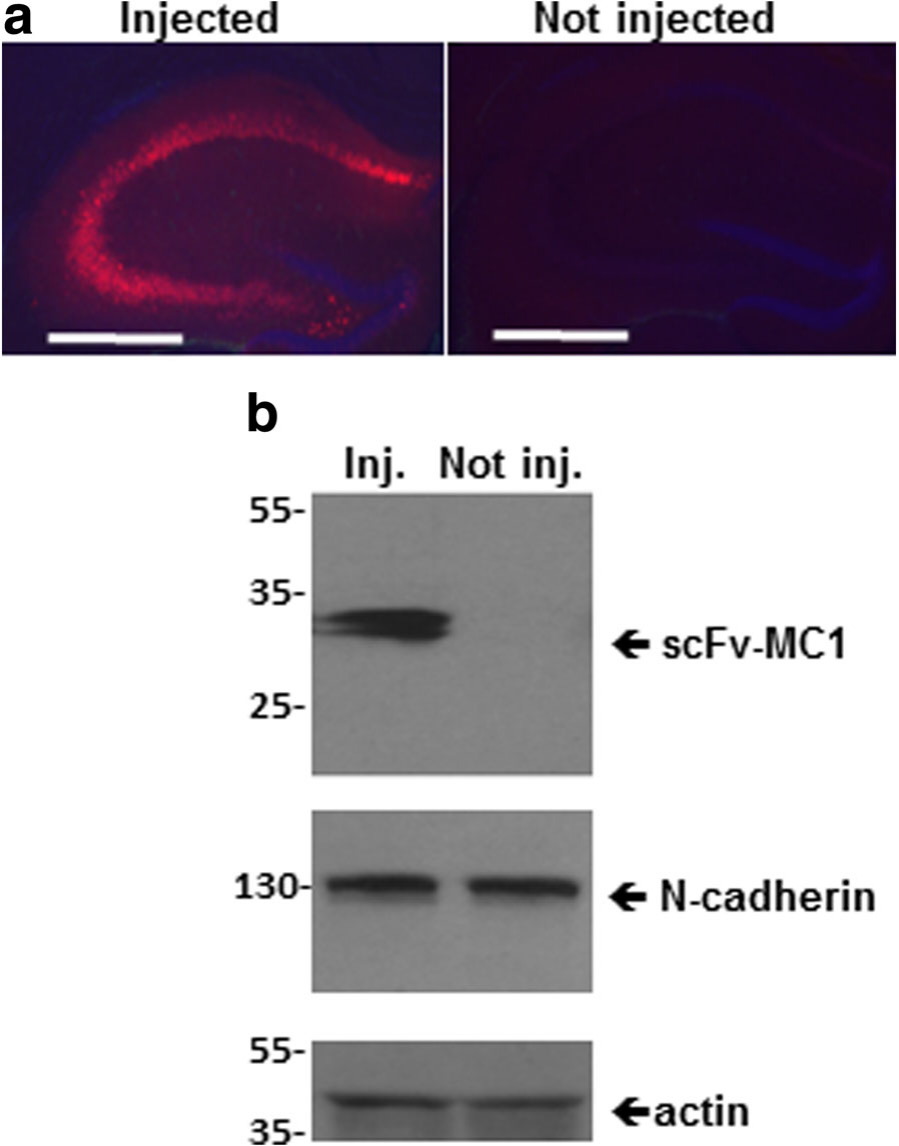
AAV5-scFv-MC1 transduction in vivo*.* **a** Representative images of JNPL3 mice brain sections, 4 weeks post injection (*n* = 6): the AAV5-scFv-MC1 injected brain showed clear expression of the recombinant antibody in the hippocampus using an anti-Myc antibody (red). Nuclei were stained with DAPI (blue). No signal was detected in the not injected brains (Zeiss Axio Imager Apotome, bar: 500 μm). **b** western blotting on hippocampus lysates of injected (Inj.) or not-injected (Not Inj.) JNPL3 mice, using anti-Myc antibody. Neuronal N-cadherin was used as a marker of neuronal integrity, and actin was used as a housekeeper

We selected the AAV5 serotype for our in vivo experiments, given its ability to transduce various brain cellular populations and providing efficient transgene expression under different promoters [10, 11, 19]. To start the in vivo study, unilateral hippocampal injection of AAV5-scFv-MC1 was performed in 3 month old JNPL3 mice, with the transgene under the expression of the strong CAG promoter. Four weeks after the injection, brains were harvested following biochemical or immunofluorescent analysis. As shown in Fig. 2a, scFv-MC1 was detected along the whole hippocampus following intracranial injection; no signal was detected in non-injected mice. Immunoblotting analysis was performed in order to confirm the scFv expression in the hippocampi: as shown in Fig. 2b, scfv-MC1 was detected as a 30KDa band in the treated animals, confirming the ability to specifically express the recombinant antibody in vivo. Of note, no reduction in the amount of neural-cadherin (N-cadherin) was detected in these brains (Fig. 2b), implying no significant neurotoxicity at this time point.

**Fig. 3:**
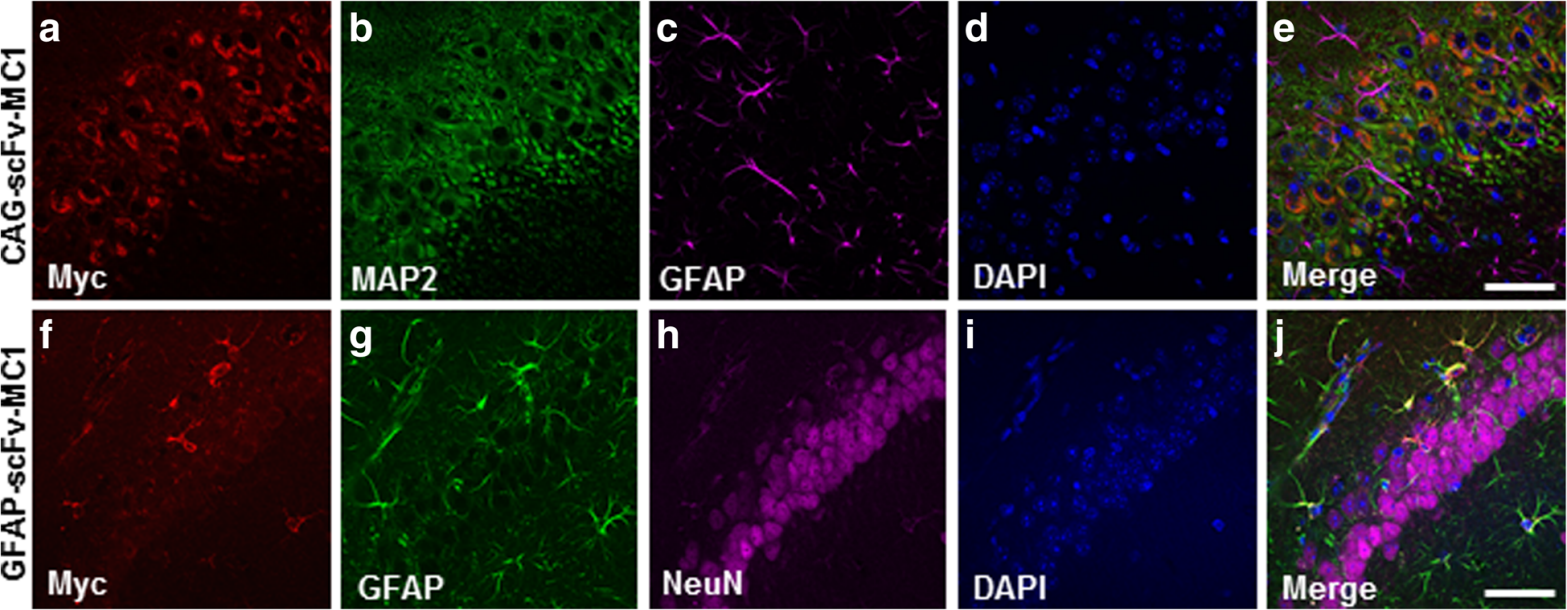
AAV5-scFv-MC1 cellular selectivity. Representative immunofluorescent confocal images of the hippocampus (CA1 quadrant) from JNPL3 mice. **a-e** When mice were injected with AAV5-CAG-scFvMC1 (n = 6), the scFv was expressed mainly in neurons: scFv-MC1 (**a**, Myc-red), MAP2-positive neurons (**b**, green), GFAP-positive astrocytes (**c**, magenta); co-localization is shown as merge of red and green (**e***,* orange/yellow). **f-j** Upon injection with AAV5-GFAP-scFvMC1 (n = 6), scFvMC1 was expressed primarily in astrocytes: scFv-MC1 (**f***,* Myc-red), GFAP-positive astrocytes (**g**, green), NeuN-positive neurons (**h**, magenta); co-localization is shown as merge of red and green (**j**, yellow). Nuclei were stained with DAPI (blue, panel **d** and **i**). Images represent maximum-intensity projection of z-stacks (Zeiss 880 confocal laser miscroscope, bar: 50 μm)

In order to optimize the in vivo system and screen for neuronal or astrocytic targeting, 3 month old JNPL3 mice were injected in the hippocampus using AAV5 expressing the transgene under the CAG or the GFAP promoters. Four weeks after the injection, mice were sacrificed and brains stained with multiple markers to track for cellular expression, showing neuronal body localization when employing the CAG promoter (Fig. 3a-e) and overall astrocytic selectivity when the GFAP promoter was used (Fig. 3f-j).

### ScFv-MC1 diffuses into the brain parenchyma

**Fig. 4:**
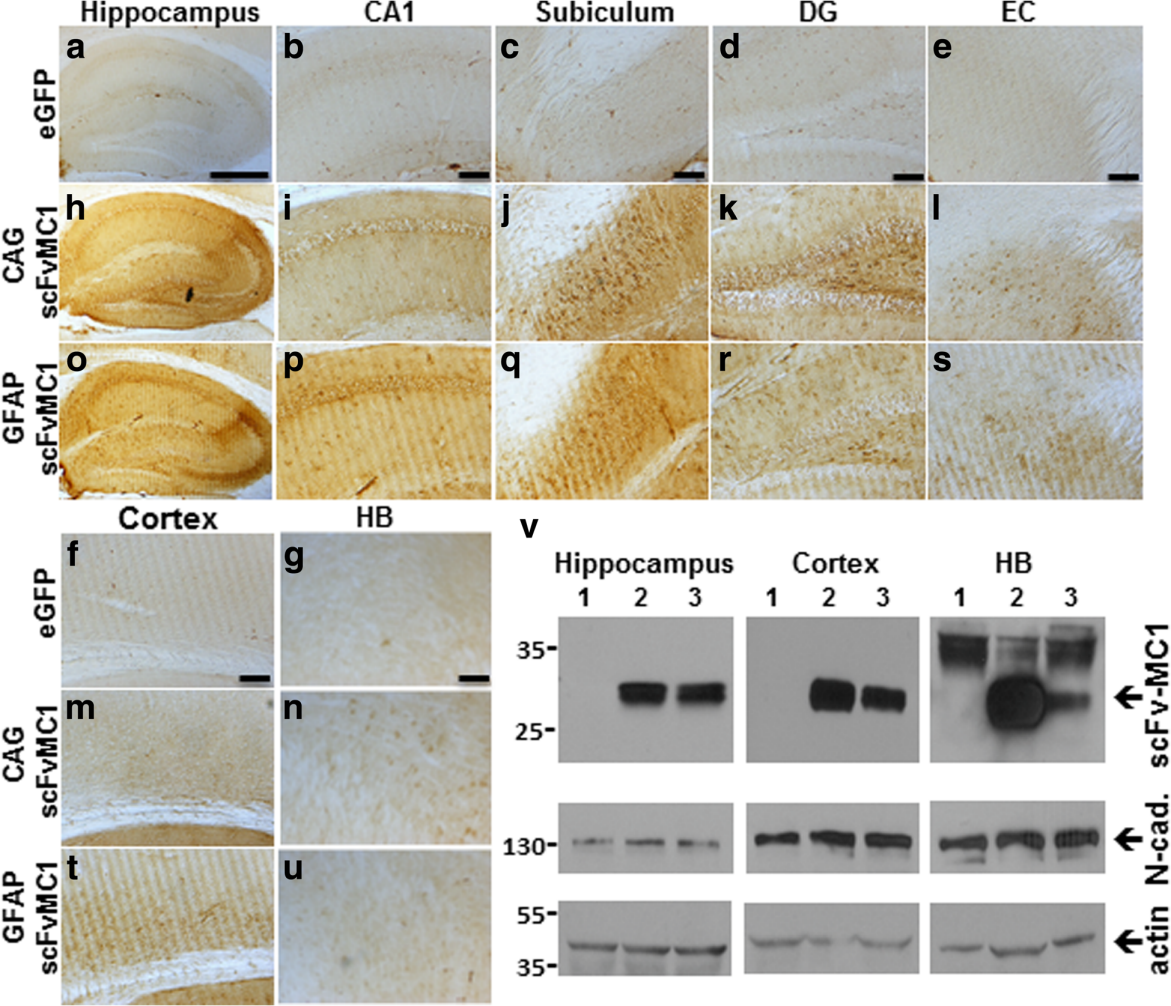
Brain parenchyma expression and diffusibility. ScFv-MC1 is expressed and released in the extracellular milieu: anti Myc-tag staining performed 4 months after the one time intracranial injection with AAV5-eGFP (**a-g**) (*n* = 15), AAV5-CAG-scFvMC1 (**h-n**) (n = 15) and AAV5-GFAP-scFvMC1 (**o-u**) (n = 15). Representative images from the whole hippocampus (Olympus BH-2 bright field microscope, bar: 500 μm), CA1, subiculum, dentate gyrus (DG), entorhinal cortex (EC), frontal cortex and HB (bar: 100 μm). The scFv is visualized as a brown signal and shows extensive expression in hippocampi from treated mice, with spreading in areas distant from the site of injection. (**v**) Immunoblot analysis of lysates from the hippocampus, cortex and hindbrain: AAV5-eGFP injected (1), AAV5-CAG-scFMC1 injected (2), AAV5-GFAP-scFvMC1 injected (3). Three representative lysates from each group of treatment were pulled together and loaded on SDS-PAGE (40μg proteins of hippocampus; 300μg proteins of cortex; 150μg proteins of HB plus HRP substrate enhancer when developing). Anti Myc-tag was used to track the scFv in the brain parenchyma; actin was used as a housekeeper and N-cadherin as a marker of neuronal integrity. Of note, immunoblots images shown represent optimal exposure samples to visualize differences within the brain areas

After accurately monitoring for scFv-MC1 expression in vivo, one-time intracranial bilateral injection of AAV5-CAG-scFv-MC1 or AAV5-GFAP-scFv-MC1 was performed in 3 months old JNPL3 mice, which were sacrificed four months later. To assess for proper scFv-MC1 release in the brain parenchyma, we tracked the recombinant tau antibody using an anti-Myc-tag antibody followed by DAB staining. When comparing the whole hippocampus from control animals (Fig. 4a) to the treated groups (Fig. 4h, o), a marked staining was observed as the result of a strong release of scFv-MC1 in the extracellular milieu. As proof of sustained production and efficient diffusion in the brain parenchyma after 4 months, we show that the recombinant antibody scFv-MC1 is detected not only at the site of injection (CA1/CA2 of hippocampus) (Fig. 4h, i, o, p**)** but also in the subiculum (Fig. 4j, q), the dentate gyrus (DG) (Fig. 4k, r), the entorhinal cortex (EC) (Fig. 4l, s) and in other areas of the cortex (Fig. 4m, t), after expression by either neurons or astrocytes. Of note, faint immunostaining is evident in the hindbrain with either systems (Fig. 4n, u). We then performed immunoblotting analysis, pulling together three representative samples from each treatment group (Fig. 4v): cortex, hippocampi and hindbrain homogenates from the scFv-MC1 injected groups show presence of the recombinant antibody, demonstrating production and/or diffusion of the antibody in the brain parenchyma. The amount of scFv-MC1 detected in the cortex and hindbrain was lower than in the site of injection (hippocampus), and required a larger amount of loading material on gels and enhanced exposure in order to get a detectable signal. The expression levels of the neural-cadherin (N-cadherin) were used as readout of neuronal integrity four months after scFv-MC1 treatment.

### MC1-tau and pThr-231 immunoreactivity are significantly reduced in the hippocampus and entorhinal cortex upon astrocytic expression of scFv-MC1

**Fig. 5:**
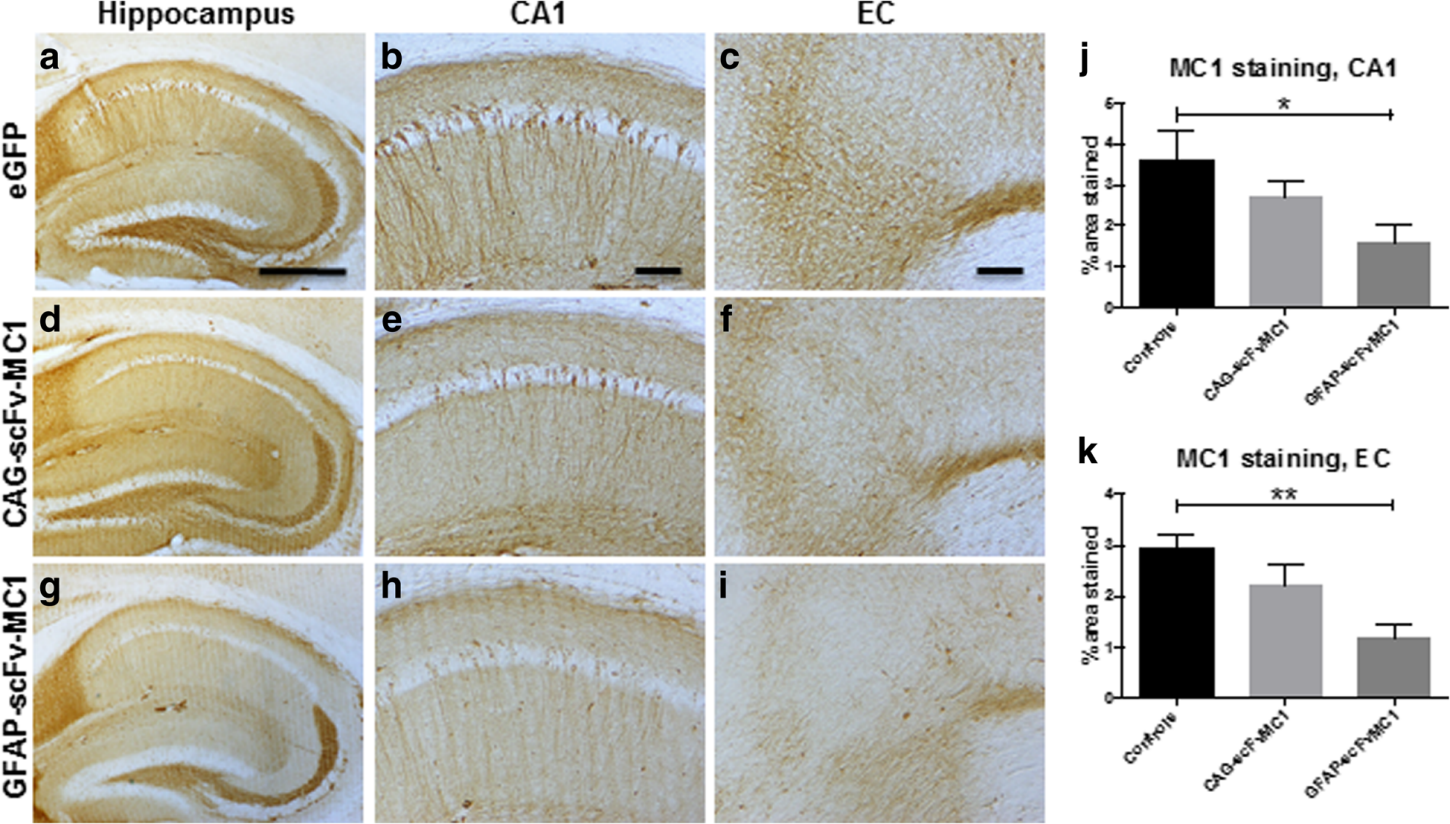
MC1-tau immunoreactivity. Representative images of MC1 staining on JNPL3 brains. **a, b, c** Non-treated mice received AAV5-eGFP injection. Treated mice were injected with AAV5-CAG-scFvMC1 **d, e, f** or AAV5-GFAP-scFvMC1 **g, h, i** (Olympus BH-2 bright field microscope; bar: 500 μm **a, d, g**; bar: 100 μm **b, c, e, f, h, i**). **j** Quantification of percentage of area stained by MC1 shows a significant reduction in the AAV5-GFAP-scFvMC1 injected group, in the CA1 region of hippocampus (*P = 0.0320 by non-parametric Kruskall-Wallis test) and **k** in the EC (**P = 0.0025 by one-way ANOVA followed by Dunnett’s post hoc test). AAV5-CAG-scFvMC1 injected mice only showed a non-significant trend reduction in MC1 staining in (**j**) CA1 (*p* = 0.3999) and in **k** EC (*p* = 0.2626). All graphs are means +/− SEM (*n* = 15 in each group of treatment)

As a proof of concept for the current immunotherapeutic approach, MC1 immunohistochemical analysis was performed (Fig. 5a-i) followed by assessment of pathology in the CA1 quadrant (Fig. 5j) of the hippocampus and the entorhinal cortex (Fig. 5k**)**. In both regions, a significant reduction of MC1-tau was detected in the astrocytic-driven expression group (GFAP-scFv) compared to the non-treated mice.

**Fig. 6:**
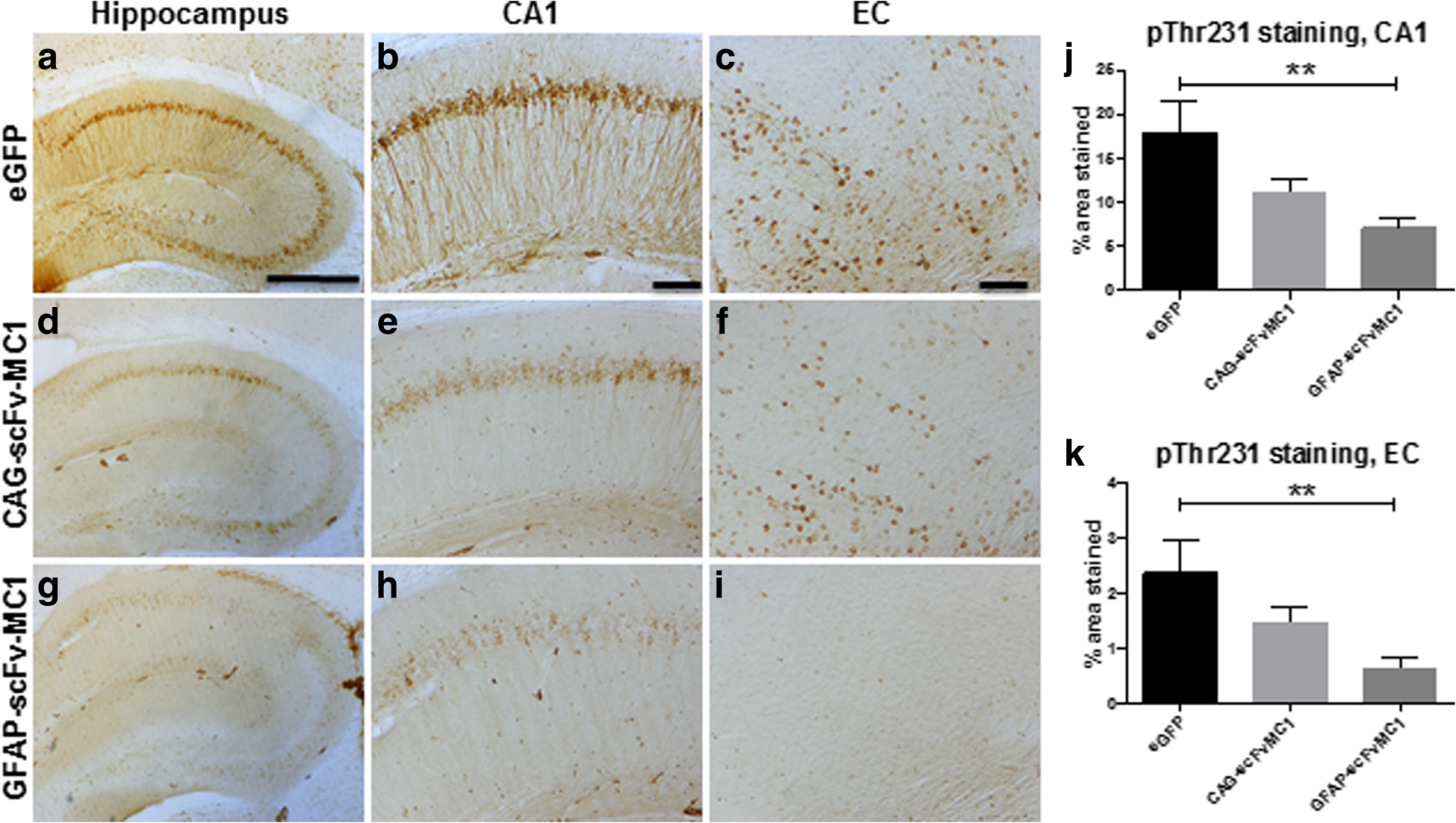
Tau phospho-immunoreactivity. Representative images of RZ3 (pThr231 tau) staining on JNPL3 brains. **a, b, c** Control mice received AAV5-eGFP injection (n = 15). Treated mice mice were injected with AAV5-CAG-scFvMC1 (n = 15) (**d, e, f**) or AAV5-GFAP-scFvMC1 (n = 15) (**g, h, i**) (bar: 500 μm **a, d, g**; bar: 100 μm **b, c, e, f, h, i**). **j** Quantification of percentage of area stained by RZ3 shows a significant reduction in the AAV5-GFAP-scFvMC1 injected group, in the CA1 region of hippocampus (**P = 0.0026 by one-way ANOVA) and (**k**) in the EC (**P = 0.0069 by one-way ANOVA). AAV5-CAG-scFvMC1 injected mice only showed a non-significant trend reduction in RZ3 staining in (**j**) CA1 (*p* = 0.0753) and in (**k**) EC (*p* = 0.1916). All graphs are means +/− SEM and were analyzed by one-way ANOVA, followed by Dunnett’s multiple comparison post hoc test, comparing every column to the eGFP injected group: **P < 0.01

In order to address the question as to whether targeting MC1-tau could affect the build-up of tau phopsho-epitopes, brain staining was performed showing a substantial reduction of phosphorylated tau at Thr-231 (RZ3) in both treatment groups compared to non-treated controls (Fig. 6a-c eGFP expression; Fig. 6d-f neuronal scFv-MC1 expression; Fig. 6g-i astrocytic scFv-MC1 expression). Quantification was performed on the CA1 quadrant of the hippocampus (Fig. 6j) and on the entorhinal cortex (EC) (Fig. 6k), showing a significant decrease in pThr-231 when the transgene was expressed under the GFAP astrocytic promoter. A trend of reduction is also evident in the CAG injected group for both MC1 and RZ3 staining (Fig. 5j-k, 6j-k). When immunohistochemical analysis was performed with PHF1 (pSer396/404) and CP13 (pSer202), no significant differences were observed in treated and non-treated animals (not shown). TUNEL and Iba1 staining have so far failed to show any evidence of cell death or differential microglia activation between treated and non-treated mice, again arguing against any toxicity (not shown).

### Phosphorylated soluble tau is significantly reduced in the hippocampus, cortex and hindbrain following scFv-MC1 injection

**Fig. 7:**
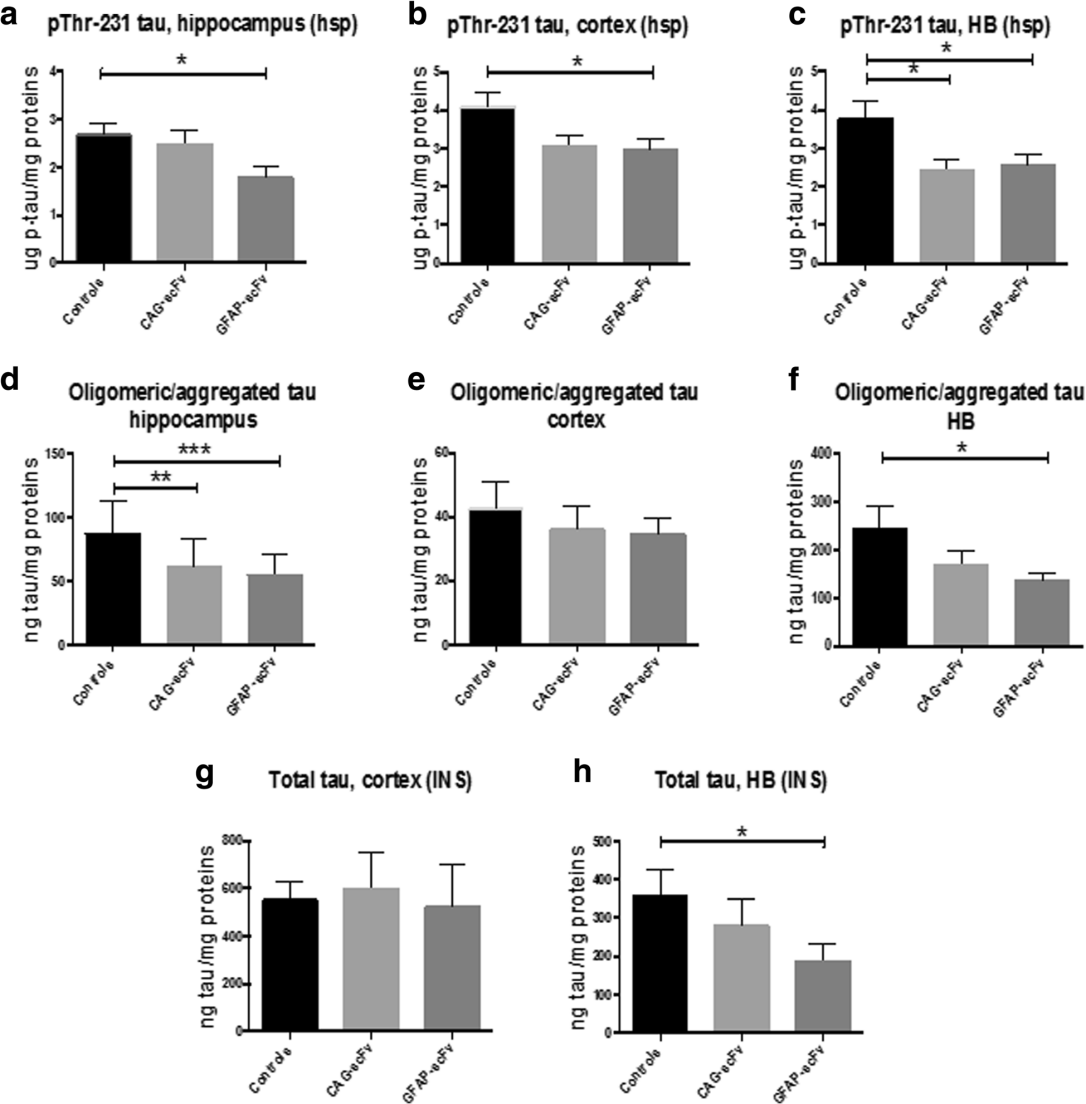
Soluble, oligomeric and insoluble tau. **a, b, c** Phosphorylated soluble tau levels (hsp: heat stable preparation) in the hippocampus, cortex and HB: pThr231 tau was significantly reduced in the hippocampus (*P = 0.036 by one-way ANOVA) and cortex (*P = 0.0351 by one-way ANOVA) when the astrocytic system was employed (GFAF-scFvMC1); in the cortex, a trend towards reduction was observed when neurons were targeted (CAG-scFvMC1, *P* = 0.0614); in the HB, pThr231 was reduced with either promoter (CAG-scFvMC1 *P = 0.0237, GFAP-scFvMC1 *P = 0.0408 by one-way ANOVA). **d, e, f** Oligomeric/aggregated tau species quantified in hippocampus, cortex and HB: oligomeric tau was significantly decreased in the hippocampus both under neuronal (**P = 0.004 by one-way ANOVA) or astrocytic expression (***P = 0.0004 by one-way ANOVA); oligomeric tau in the HB was significantly reduced only when using the GFAP-scFvMC1 system (*P = 0.0444 by one-way ANOVA); no modulation of oligomeric tau species was found in the cortex. **g, h** Insoluble tau (INS) was analyzed in the cortex and HB: no changes were detected in the cortex, while the HB showed a significant reduction under the expression of the GFAP promoter (*P = 0.0378 by non-parametric Kruskall-Wallis test). All graphs are means +/− SEM (n = 15 in each group of treatment)

Three different cerebral regions were dissected upon sacrifice: hippocampus, cortex and hindbrain. Biochemical analysis of soluble tau, by ELISA, showed reduction of phosphorylated tau at the Thr231 residue. In the hippocampus (Fig. 7a), soluble phospho-tau Thr231 was significantly reduced when the astrocytic promoter GFAP was employed. Consistent with the previously anticipated parenchymal diffusion of scFv-MC1 from the site of injection, tau phosphorylated at Thr231 was significantly reduced in the cortex (Fig. 7b) and hindbrain’s soluble fractions (Fig. 7c), with the GFAP promoter displaying again the more significant effect. Cortical total soluble tau was reduced with either promoter (Additional file 1: Figure S1d), as seen before in the whole forebrain fractions [17].

### Oligomeric/aggregated tau is significantly reduced in the hippocampus and hindbrain upon astrocytic expression of the scFv-MC1

Taking advantage of our tau mono-ELISA [22], we have analyzed the amount of oligomeric/aggregated species in the lysates belonging to the different brain areas. Tau aggregated forms in the hippocampus were significantly decreased when the scFv-MC1 was under neuronal or astrocytic expression (Fig. 7d), again with the astrocytic promoter being more efficient. In the cortex, a trend towards reduction is seen (Fig. 7e). Aggregated tau in the hindbrain was significantly reduced only when targeting the astrocytic population to release scFv-MC1 (Fig. 7f).

### Insoluble tau is reduced in the HB and cortex

Analysis of the insoluble fraction was performed on cortex and hindbrain. In our protocol, due to the technical requirement to obtain the insoluble tau fraction, i.e. the need for a sufficient amount of tissue, this analysis was not performed on the hippocampus. Instead, as shown above, the tau mono-ELISA was used as a functional surrogate of insoluble tau.

As shown in Fig. 7h, a reduction of total insoluble tau in the hindbrain was seen in the astrocytic expression group. While no effect was detected on total insoluble tau in cortex (Fig. 7g), analysis of different phosphorylation residues showed a significant decrease of insoluble cortical tau phosphorylated at Thr231, together with a trend toward reduction of the other phospho-epitopes in both areas (Additional file 1: Figure S2a-f), again more pronounced with the GFAP promoter.

## Discussion

In an effort to overcome the limitations of conventional immunotherapy, including the need for multiple administrations, we engineered scFv-MC1, cloned it into an AAV vector, and delivered it to the brain via one-time bilateral intracranial injection, to achieve sustained production and release of the antibody. Here we show that: 1) scFv-MC1 shares the same specificity of the parent MC1 antibody; 2) AAV5-vectored scFv-MC1 can be efficiently expressed by neurons or astrocytes; 3) scFv-MC1 can spread from the site of injection to adjacent brain structures; 4) scFv-MC1 is able to reduce/prevent tau pathology in JNPL3 adult mice, even in sites distant from injection, with efficacy on different tau species (soluble, oligomeric, insoluble), and more notably with astrocytic production.

We selected two different cellular populations to express and secrete the engineered scFv-MC1. In our study, the astrocytic system was overall more efficient at reducing pathological tau species in adult JNPL3 mice. The effect was not directly related to the amount of scFv released in the extracellular milieu. In fact, both systems expressed a comparable amount of recombinant antibody in the hippocampus and cortex. In the hindbrain, a location far from the injection site, we detected more scFv-MC1 when using the neuronal system, by biochemical analysis. It is still unclear whether this is due to a better diffusivity of the antibody to the HB or to a higher expression by the stronger CAG promoter. A higher presence of scFv-MC1 in the HB did not result in more robust reduction of tau pathology in this area. Further studies of the expression/diffusion of scFv through the brain parenchyma are needed to understand this complex dynamic.

In this study, we performed an accurate and extensive biochemical analysis of soluble, oligomeric and insoluble tau species in the hippocampus, cortex and HB, showing a consistent pattern of reduction of pathological tau upon treatment with our recombinant antibody. A decrease in soluble pThr231 was common to the three brain areas analyzed upon astrocytic expression of the scFv-MC1 (hippocampus: − 35%; cortex: -25%; HB: -33%), while no major effects were detected on other soluble tau/phosphorylation residues (CP13: Ser202; PHF1: Ser396–404) (Additional file 1: Figure S1). Supporting these data, we found that the pThr231 immunoreactivity in the hippocampus and EC was significantly reduced in the injected mice (hippocampus: − 60% circa; EC: − 70% circa), consistent with the reduced tau-MC1 immunostaining (hippocampus: − 60% circa; EC: − 70% circa). Immunostaining using CP13 and PHF1 confirmed the previous findings (not shown). We believe that the general selectivity for the Thr231 epitope is related to the fact that when our treatment first begins we are targeting soluble tau and its early modifications, e.g. Thr231. Upon binding soluble MC1-tau, the recombinant antibody appears to be able to selectively reduce/prevent the buildup of soluble pThr231-tau as well.

Soluble oligomeric tau represents a transition state between physiological soluble and pathological insoluble tau aggregates. To detect oligomeric tau, we used a mono-ELISA [22]. This assay allows the detection of aggregated tau species, ranging from dimers to larger aggregates, and represents a good marker of progression towards the formation of neurofibrillary pathology in tau transgenic animal models. The percentage of decrease of oligomeric tau was calculated to correspond to a 40% and 45% reduction, in the hippocampus and HB respectively. No effect was detected in the cortex. Since these species mostly belong to the soluble pool of tau, we estimated, in our control group, the percentage of oligomeric species relative to the whole tau soluble fraction for each separate brain region: 1% in the hippocampus, 0.5% in the cortex and 3.5% in the HB. We believe that this component resembles the soluble tau species recently described by Takeda et al. [46]. This group has shown that PBS-soluble phosphorylated high-molecular-weight tau species are axonally transported and passed on to synaptically connected neurons. These species have been described as globular but non-fibrillar structures, accounting for only a small fraction of all soluble tau species, estimated at < 10% in mutant-tau rTg4510, similar to our data. Interestingly, these structures also have a pathological misfolded conformation, positive for the Alz50/MC1 antibody [46]. We speculate that scFv-MC1 binds these soluble globular aggregated structures, lowering their total amount, most likely in the extracellular milieu, hence blocking their further spreading.

As for oligomeric tau, the total insoluble fraction was unchanged in the cortex and reduced in the HB. The 48% reduction of insoluble tau in the HB overlaps the 45% reduction of the oligomeric species in the same area. Hence, in this “prevention protocol”, we assume that the decreased accumulation of fibrillar aggregates is a consequence of the reduced build-up of the oligomeric species. This assumption is supported by the fact that tau levels in serum are unchanged (Additional file 1: Figure S3), making the peripheral sink hypothesis, as a mechanism of clearance of the insoluble aggregates, unlikely. Considering the data on hippocampal oligomeric/aggregated tau (DA9/DA9 assay), we speculate that a similar reduction of insoluble tau is present in this area as well. More studies are needed to clarify if, by employing a “therapeutic protocol” treating mice at more advanced stages, we will achieve clearance of established tau pathology.

In our estimate, the brain concentration of scFv-MC1 obtained by intracranial injection is in good excess to the amount of tau to target. Nevertheless, our results show a partial reduction of build-up, or clearance, of tau pathological species. Simply aiming at one tau epitope might not be enough to reduce all pathology. Also, as suggested in the neuronal delivery system, increasing the “dose” of scFv-MC1 in mice might not be the solution, in this tau over-expressing model. Targeting multiple epitopes, e.g. adding total tau or other phosphorylation specific antibodies to scFv-MC1, may result in a more effective synergistic approach. More studies are needed to explore this possibility. Of note, scFv-MC1 was not detected in serum, by neither immunoblotting or ELISA (data not shown), as already reported by others [26].

The usefulness of adeno-associated viruses as a gene delivery system has been extensively used in clinical trials, including via intracranial administration [31, 34, 36, 38, 41, 42, 48]. Even though they are generally known for low levels of immunogenicity [54], AAV’s efficacy could be limited if the viral transduction per se led to a significant alteration of the normal function of the tissue under observation, or to the activation of the host immune system with the production of neutralizing antibodies (NAB) directed to the transgene [15, 20, 37, 43, 54]. In our study, the levels of inflammation (not shown) and neuronal integrity were assessed, and are negligible. Also, antibodies directed to scFv-MC1 were found in plasma of few animals upon sacrifice (Additional file 1: Figure S4a), suggesting an immunogenic response to the scFv (or a fragment thereof). However, considered the success at reducing soluble, oligomeric/aggregated and insoluble tau in different brain regions, together with the correlation analysis data generated (Additional file 1: Figure S4b, c), we believe that the anti-scFv-MC1 do not affect efficacy, hence are so far irrelevant in this study.

## Conclusions

Overall, our results are encouraging for tau-directed scFv immunotherapy, and are a good premise for translational applications. AAV peripheral administration might be a viable option, if penetration of the BBB or constitution of a peripheral scFv “factory” are achieved. These will be the scope of our further studies.

## Additional file

Additional file 1: **Figure S1.** Total and phosphorylated soluble tau in hippocampus, cortex and HB**.** (**a, b, c**) In the hippocampus, total tau was not modulated, as well as the pSer202 and pSer396–404 phospho-epitopes. (**d, e, f**) In the cortex, a significant reduction of total soluble tau was reached with both the CAG (*P = 0.0325 by one-way ANOVA) and the GFAP promoter (**P = 0.006 by one-way ANOVA). (**g, h, i)**. In the HB, phosphorylation at Ser202 was significantly decreased in the GFAP-scFMC1 treatment group (*P = 0.0112 by one-way ANOVA). Error bars indicate means +/− SEM. **Figure S2.** Phosphorylated insoluble tau (INS) in cortex and HB**.** (**a, b, c**) Phosphorylation levels of insoluble tau in the cortex: the pThr231 residues showed a significant reduction (*P = 0.0398 by non-parametric Kruskall-Wallis test) in the GFAP expressed group, while pSer202 (*P* = 0.0685) and pSer396–404 (*P* = 0.0649) showed a non-significant trend towards reduction in the same treatment cohort. (**d, e, f**) In the HB, no significant decrease in phosphorylated insoluble tau was detected, with a trend to reduction on pSer396–404 in the GFAP-scFv treated group (*P* = 0.0768). Error bars indicate means +/− SEM. **Figure S3.** Tau levels in serum. At sacrifice, total tau was analyzed in serum (ng/ml), showing no significant difference between groups. **Figure S4.** Antibodies directed to the scFv-MC1, in serum. (**a**) At sacrifice, few animals in both groups of treatment were positive for the presence of anti-scFv-MC1. (**b**) Linear regression analysis: in the neuronal expression group (CAG promoter) no significant correlation was detected between the concentration of anti-scFvMC1 antibodies in the serum (ug/ml) and the concentrations of the oligomeric/aggregated tau in hippocampus (r^2^ = 0.095). (**c**) In the astrocytic expression group (GFAP promoter) the same analysis was performed, again with no significant correlation between anti-scFvMC1 antibodies and oligomeric/aggregated tau in hippocampus (r^2^ = 0.018). (PDF 851 kb)
