# Supplementary material for: Anti-tau conformational scFv MC1 antibody efficiently reduces pathological tau species in adult JNPL3 mice
Source: Acta Neuropathol Commun. 2018 Aug 22;6:82. doi: 10.1186/s40478-018-0585-2 (PMC6103963; doi:10.1186/s40478-018-0585-2)
Supplement: Supplementary file 1 — Figure S1. Total and phosphorylated soluble tau in hippocampus, cortex and HB. (a, b, c) In the hippocampus, total tau was not modulated, as well as the pSer202 and pSer396–404 phospho-epitopes. (d, e, f) In the cortex, a significant reduction of total soluble tau was reached with both the CAG (*P = 0.0325 by one-way ANOVA) and the GFAP promoter (**P = 0.006 by one-way ANOVA). (g, h, i). In the HB, phosphorylation at Ser202 was significantly decreased in the GFAP-scFMC1 treatment group (*P = 0.0112 by one-way ANOVA). Error bars indicate means +/− SEM. Figure S2. Phosphorylated insoluble tau (INS) in cortex and HB. (a, b, c) Phosphorylation levels of insoluble tau in the cortex: the pThr231 residues showed a significant reduction (*P = 0.0398 by non-parametric Kruskall-Wallis test) in the GFAP expressed group, while pSer202 (P = 0.0685) and pSer396–404 (P = 0.0649) showed a non-significant trend towards reduction in the same treatment cohort. (d, e, f) In the HB, no significant decrease in phosphorylated insoluble tau was detected, with a trend to reduction on pSer396–404 in the GFAP-scFv treated group (P = 0.0768). Error bars indicate means +/− SEM. Figure S3. Tau levels in serum. At sacrifice, total tau was analyzed in serum (ng/ml), showing no significant difference between groups. Figure S4. Antibodies directed to the scFv-MC1, in serum. (a) At sacrifice, few animals in both groups of treatment were positive for the presence of anti-scFv-MC1. (b) Linear regression analysis: in the neuronal expression group (CAG promoter) no significant correlation was detected between the concentration of anti-scFvMC1 antibodies in the serum (ug/ml) and the concentrations of the oligomeric/aggregated tau in hippocampus (r2 = 0.095). (c) In the astrocytic expression group (GFAP promoter) the same analysis was performed, again with no significant correlation between anti-scFvMC1 antibodies and oligomeric/aggregated tau in hippocampus (r2 = 0.018). (PDF 851 kb) [file 40478_2018_585_MOESM1_ESM.pdf]

Anti-tau conformational scFv MC1 antibody efficiently reduces pathological tau species in adult JNPL3 mice

Acta Neuropathologica Communications

Francesca Vitale[1], Luca Giliberto[1], Santiago Ruiz[1], Kristen Steslow[1], Philippe Marambaud[1], Cristina d'Abramo[1].

[1]Litwin-Zucker Center for Research in Alzheimer's disease

The Feinstein Institute for Medical Research

Northwell Health

350 Community Drive, Manhasset

11030 NY, USA

Corresponding author: Cristina d'Abramo, PhD

Address: Litwin-Zucker Center for Research in Alzheimer's disease

The Feinstein Institute for Medical Research

350 Community Dr. Manhasset, NY11030

Email: cdabramo@northwell.edu

Tel: +1-516-562-3495

Fax: +1-516-487-3792

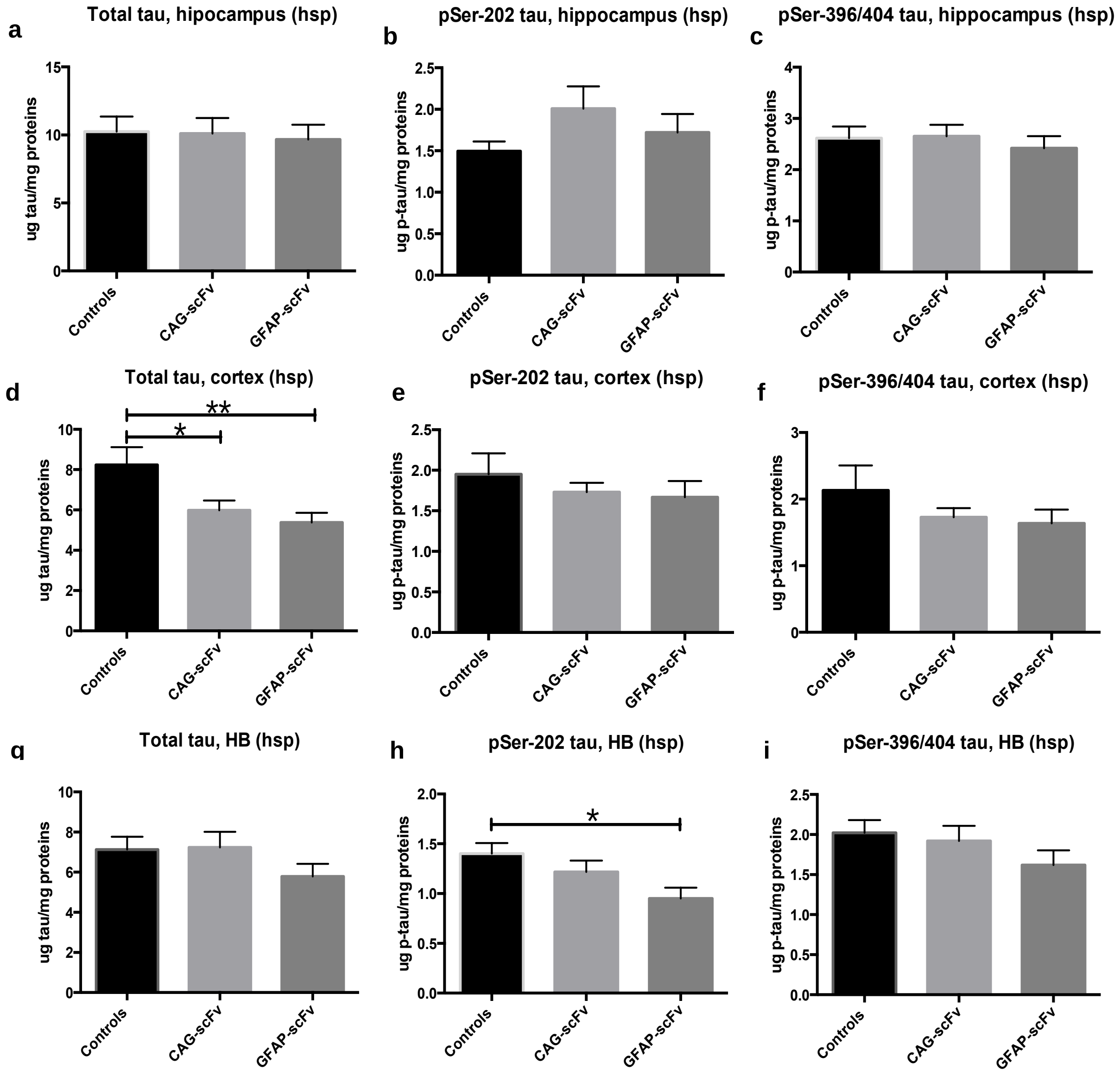

Fig. S1 Total and phosphorylated soluble tau in hippocampus, cortex and HB**.** (**a, b, c**) In the hippocampus, total tau was not modulated, as well as the pSer202 and pSer396-404 phospho-epitopes. (**d, e, f**) In the cortex, a significant reduction of total soluble tau was reached with both the CAG (*P=0.0325 by one-way ANOVA) and the GFAP promoter (**P=0.006 by one-way ANOVA). (**g, h, i**). In the HB, phosphorylation at Ser202 was significantly decreased in the GFAP-scFMC1 treatment group **(***P=0.0112 by one-way ANOVA). Error bars indicate means +/- SEM

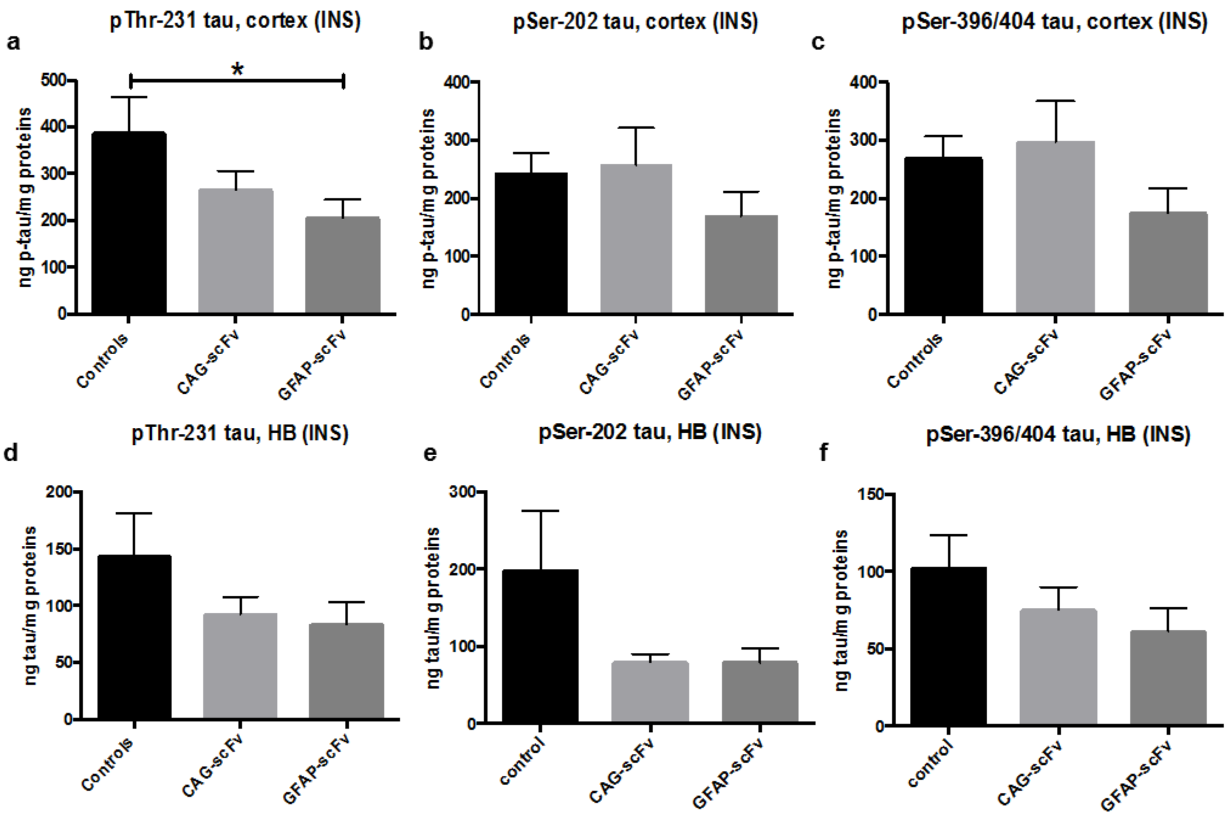

Fig. S2 Phosphorylated insoluble tau (INS) in cortex and HB**.** (**a, b, c**) Phosphorylation levels of insoluble tau in the cortex: the pThr231 residues showed a significant reduction (*****P=0.0398 by non-parametric Kruskall-Wallis test) in the GFAP expressed group, while pSer202 (P=0.0685) and pSer396-404 (P=0.0649) showed a non-significant trend towards reduction in the same treatment cohort. (**d, e, f**) In the HB, no significant decrease in phosphorylated insoluble tau was detected, with a trend to reduction on pSer396-404 in the GFAP-scFv treated group (P=0.0768). Error bars indicate means +/- SEM

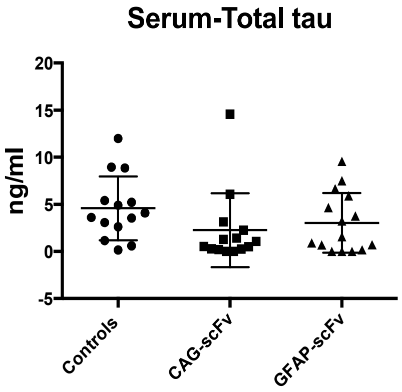

Fig. S3 Tau levels in serum. At sacrifice, total tau was analyzed in serum (ng/ml), showing no significant difference between groups

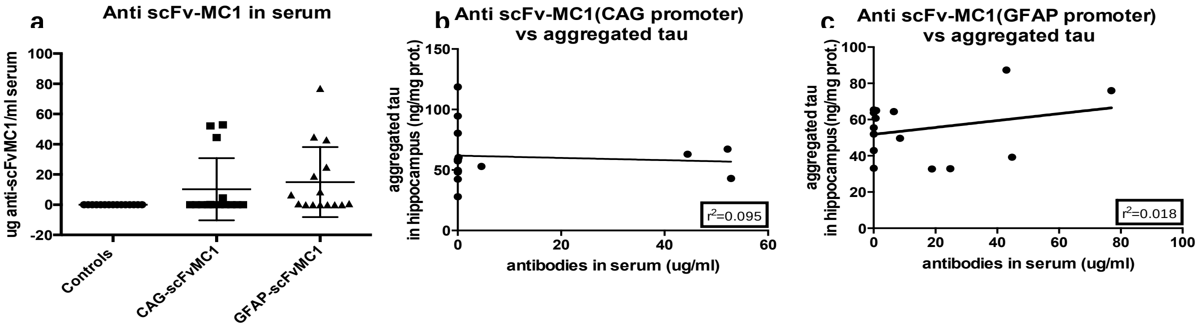

Fig. S4 Antibodies directed to the scFv-MC1, in serum. (**a**) At sacrifice, few animals in both groups of treatment were positive for the presence of anti-scFv-MC1. (**b**) Linear regression analysis: in the neuronal expression group (CAG promoter) no significant correlation was detected between the concentration of anti-scFvMC1 antibodies in the serum (ug/ml) and the concentrations of the oligomeric/aggregated tau in hippocampus (r^2= 0.095). (**c**) In the astrocytic expression group (GFAP promoter) the same analysis was performed, again with no significant correlation between anti-scFvMC1 antibodies and oligomeric/aggregated tau in hippocampus (r^2= 0.018)
